# Educational and employment outcomes associated with childhood traumatic brain injury in Scotland: A population-based record-linkage cohort study

**DOI:** 10.1371/journal.pmed.1004204

**Published:** 2023-03-28

**Authors:** Meghan J. Visnick, Jill P. Pell, Daniel F. Mackay, David Clark, Albert King, Michael Fleming

**Affiliations:** 1 School of Health and Wellbeing, University of Glasgow, Glasgow, United Kingdom; 2 Public Health Scotland, Edinburgh, United Kingdom; 3 ScotXed, Scottish Government, Edinburgh, United Kingdom; London School of Hygiene and Tropical Medicine, UNITED KINGDOM

## Abstract

**Background:**

Traumatic brain injury (TBI) is a leading cause of death and disability among young children and adolescents and the effects can be lifelong and wide-reaching. Although there have been numerous studies to evaluate the impact of childhood head injury on educational outcomes, few large-scale studies have been conducted, and previous research has been limited by issues of attrition, methodological inconsistencies, and selection bias. We aim to compare the educational and employment outcomes of Scottish schoolchildren previously hospitalised for TBI with their peers.

**Methods and findings:**

A retrospective, record-linkage population cohort study was conducted using linkage of health and education administrative records. The cohort comprised all 766,244 singleton children born in Scotland and aged between 4 and 18 years who attended Scottish schools at some point between 2009 and 2013. Outcomes included special educational need (SEN), examination attainment, school absence and exclusion, and unemployment. The mean length of follow up from first head injury varied by outcome measure; 9.44 years for assessment of SEN and 9.53, 12.70, and 13.74 years for absenteeism and exclusion, attainment, and unemployment, respectively. Logistic regression models and generalised estimating equation (GEE) models were run unadjusted and then adjusted for sociodemographic and maternity confounders. Of the 766,244 children in the cohort, 4,788 (0.6%) had a history of hospitalisation for TBI. The mean age at first head injury admission was 3.73 years (median = 1.77 years). Following adjustment for potential confounders, previous TBI was associated with SEN (OR 1.28, CI 1.18 to 1.39, *p* < 0.001), absenteeism (IRR 1.09, CI 1.06 to 1.12, *p* < 0.001), exclusion (IRR 1.33, CI 1.15 to 1.55, *p* < 0.001), and low attainment (OR 1.30, CI 1.11 to 1.51, *p* < 0.001). The average age on leaving school was 17.14 (median = 17.37) years among children with a TBI and 17.19 (median = 17.43) among peers. Among children previously admitted for a TBI, 336 (12.2%) left school before age 16 years compared with 21,941 (10.2%) of those not admitted for TBI. There was no significant association with unemployment 6 months after leaving school (OR 1.03, CI 0.92 to 1.16, *p* = 0.61). Excluding hospitalisations coded as concussion strengthened the associations. We were not able to investigate age at injury for all outcomes. For TBI occurring before school age, it was impossible to be certain that SEN had not predated the TBI. Therefore, potential reverse causation was a limitation for this outcome.

**Conclusions:**

Childhood TBI, sufficiently severe to warrant hospitalisation, was associated with a range of adverse educational outcomes. These findings reinforce the importance of preventing TBI where possible. Where not possible, children with a history of TBI should be supported to minimise the adverse impacts on their education.

## Abstract 2

Meghan J Visnick and colleagues compare the educational and employment outcomes of Scottish schoolchildren previously hospitalised for traumatic brain injury with their peers.

## Author summary

**Why was this study done?:**

- Traumatic brain injury (TBI) is a leading cause of morbidity and mortality among young children and adolescents and has wide-reaching consequences spanning health and education.
- Most previous studies of childhood TBI and educational outcomes have been small-scale.

**What did the researchers do and find?:**

- We identified a population-wide cohort of Scottish schoolchildren attending school in Scotland between 2009 and 2013 and linked their school data to hospitalisation data to identify children previously admitted to hospital for TBI (*n* = 4,788) and compare subsequent educational outcomes with peers (*n* = 761,456).
- Compared to their peers, children previously hospitalised for TBI had greater special educational need (SEN), more school absences, more exclusions, and lower academic attainment post injury. However, no significant differences were observed in post school unemployment.

**What do these findings mean?:**

- Children with a history of TBI should be provided with support and monitoring to minimise adverse impacts on their educational outcomes.
- It was impossible to be certain that some SEN had not predated TBI resulting in potential reverse causation and possible overestimation of the strength of the observed association between TBI and SEN. This specific finding should therefore be interpreted with caution.

## Introduction

In the United Kingdom, 280 children per 100,000 are admitted to hospital annually for traumatic brain injury (TBI) [1]. TBI is a leading cause of death and disability in childhood and increases the risk of psychiatric disorders [2–7], suicide [8], criminal behaviour, and incarceration in later life [9,10]. Childhood is an important period for brain development and learning and, while 80% to 90% of childhood TBI cases are mild [11], moderate and severe injuries can result in physical, psychosocial, cognitive, emotional, and behavioural deficits that impair normal development and the ability to participate in home, school, and community activities [12–15].

A meta-analysis of 28 studies with follow-up between 6 months and 2 years reported no significant long-term effects on cognitive outcomes following mild TBI, but persistent deficits in intellectual ability, memory, and attention following moderate and severe TBI, as well as slower rates of cognitive development and deterioration in executive function [16]. The deficits resulting from childhood TBI can require lifelong support [11,16–19], reducing the quality of life of both the affected individual and their caregivers [20–22]. The aim of this study was to use record linkage of Scottish administrative data to investigate a range of educational outcomes following hospitalised TBI in childhood. We hypothesise that children who have been hospitalised for TBI will have poorer educational outcomes compared to their peers.

## Methods

This study is reported as per the Strengthening the Reporting of Observational Studies in Epidemiology (STROBE) guideline (S1 STROBE Checklist). While we did not publish an analysis plan, our analyses were planned before the research team accessed any data. Data were extracted in June 2015 as part of a wider linkage project [23] and this specific study commenced in February 2021 utilising the same data. A Scotland-wide cohort was constructed by linking, at an individual level, 2 health sector databases held by Public Health Scotland to 3 education sector databases held by the Scottish Exchange of Educational Data (ScotXed), using a two-step process involving probabilistic and deterministic matching. This linkage methodology has been described in detail elsewhere [23] and shown to be 99% accurate for singleton births [24]. The cohort comprised singleton children who were born in Scotland and attended a Scottish school, aged 4 to 18 years, at some point between 2009 and 2013. Inclusion was restricted to singleton children because of difficulties linking same sex multiple births to the correct education records.

The Scottish Morbidity Record 02 (SMR02) collects maternal, obstetric, and neonatal data on all pregnancy-related admissions. SMR01 records acute hospital admissions, including dates of admission and discharge and International Classification of Diseases (ICD-10) diagnostic codes. The annual School Pupil Census collects data at the start of each school year from all local authority maintained primary, secondary, and special schools in Scotland. The data collected include demographic information, stage of education, and record of special educational need (SEN). Attendance data are recorded biennially on school absences (due to illness or prior arrangement) and school exclusions. They are collected prospectively then appended to the School Pupil Census at the end of the school year. The Scottish Qualifications Authority (SQA) collects grades attained in national examinations undertaken over the last 3 years of school attendance. The School Leaver Destination Survey is self-completed by 99% of school leavers 6 months after leaving school [25]. Destination was classified as being in paid or voluntary employment, training, further or higher education, or being unemployed.

In the Scottish education system, children attend 7 years of primary school education (P1-P7) from ages 4/5 through 10/11 followed by 4 or 5 mandatory years of secondary education (S1-S4/S5) from ages 11/12 until their 16th birthday. Children can leave school when they turn 16 but many go on to complete the last 2 years of secondary school (S5 and S6). The outcomes analysed were: record of all-cause SEN, cause-specific SEN, school absences, school exclusions, low attainment in examinations, and unemployment. There is a statutory requirement for schools to record SEN, defined as a requirement for additional support, beyond that normally provided to children of the same age. All-cause SEN was defined as SEN due to learning disability; learning difficulty; sensory impairment; physical or motor impairment; communication problems; autistic spectrum disorder; physical health problems; mental health problems; and/or social, emotional, or behavioural difficulties. A child can have more than 1 cause recorded. Overall level of attainment over the last 3 years of secondary school (S4-S6) was derived from the total number of examination grades attained at each level of the Scottish Credit and Qualifications Framework (SCQF) [26]. Different levels of exam and grade combination correspond to different SCQF levels. The overall scoring system takes account of external examinations at different levels that can either be taken chronologically in the same children or at the same age by 2 different children. These exams are normally taken between 14 and 18 years of age. For this study, overall attainment was dichotomised as low attainment (≥1 awards corresponding to SCQF level 2, ≥5 awards corresponding to SCQF level 3, ≥2 but ≤7 awards corresponding to SCQF level 4, or >0 but ≤4 awards corresponding to SCQF level 5) or high attainment (>7 awards corresponding to SCQF level 4, ≥5 awards corresponding to SCQF level 5, ≥3 awards corresponding to SCQF level 6, or ≥1 award corresponding to SCQF level 7). Destination 6 months after leaving school was collapsed into a dichotomous variable: in education/employment/training or unemployed. The attainment and unemployment analyses were restricted to school leavers. Attainment analyses were additionally restricted to children who had either sat, or were of an age eligible to sit, exams across all 3 school stages S4, S5, and S6 during the study period. These methods, which ensured that potential follow-up time to attain academic awards was comparable across different children, are described elsewhere [23]. The number of records and pupils excluded at each stage of data cleaning and used to investigate each outcome are detailed in S1 Fig.

The exposure of interest was TBI which was defined as admission to hospital with a relevant International Classification of Diseases (ICD) head injury code in any position: skull fracture (ICD-9 800–801, 803, 804; ICD-10 S02.0, S02.1, S02.3, S02.7 to S02.9), intracranial injury (ICD-9 850–854; ICD-10 S06.0 to S06.90), or unspecified head injury (ICD-10 S09.90). To meet the definition of being a previous TBI admission, the first TBI admission had to occur prior to available education outcome data for that pupil. Codes relating to injuries of the nose, ear, and chin were not included in the definition because they are unlikely to cause TBI in isolation. As a sensitivity analysis, the analyses were re-run excluding children whose hospital admissions for TBI were coded as concussion (ICD-9 850; ICD-10 S06.0) which is, by definition, synonymous with mild TBI.

For each outcome, 3 models were constructed: univariate, partially adjusted (adjusted for sociodemographic covariates), and fully adjusted (adjusted for sociodemographic and maternity covariates). We adjusted for maternity covariates because previous studies have demonstrated an association between maternity factors and long-term cognitive sequelae including SEN [27–29]. Pupil sociodemographic covariates comprised: age (derived from date of birth and date of School Pupil Census), sex, ethnic group, and current area deprivation quintile category. Deprivation quintile category was based on the Scottish Index of Multiple Deprivation (SIMD), derived from the postcode of residence recorded on the School Pupil Census. SIMD is a composite measure covering 7 domains: income, employment, education, health, access to services, crime, and housing [30]. Maternity covariates comprised: maternal age, marital status, smoking status, parity, mode of delivery, gestational age at delivery, sex- and gestation-specific birth weight centile, and 5-min Apgar score.

Attainment and unemployment were analysed using binary logistic regression models.

Children had serial measurements of SEN, absenteeism, and exclusion over more than 1 School Pupil Census, violating the assumption of independent observations. Therefore, these outcomes were analysed using generalised estimating equation (GEE) models to take account of correlations between measures recorded for the same pupil across different census years [31]. All-cause SEN and cause-specific SEN were binary outcomes investigated using GEE models with a binomial distribution and logit link function. School absences and exclusions were count outcomes investigated using GEE models with a negative binomial distribution and log-link function with the number of possible attendances included as an offset to adjust for different exposure times. Correlation structures were chosen based on the quasi-likelihood under the independence model criterion (QIC) values [32]. The correlation structure with the lowest QIC was deemed the most appropriate.

Analyses were conducted using R version 4.2.0 and Stata MP version 17. Approval for the study was granted by the Public Benefit and Privacy Panel of Public Health Scotland (reference 1920–0144).

### Approvals

The NHS West of Scotland Research Ethics Service confirmed that formal NHS ethics approval was not required since the study involved linkage of routinely collected data with an acceptably negligible risk of identification. The study was approved by the Public Benefit and Privacy Panel of NHS Public Health Scotland. A data processing agreement was drafted between Glasgow University and Public Health Scotland and a data sharing agreement between Glasgow University and ScotXed. The linked data were anonymised, then stored and analysed within the national safe haven.

### Public and patient involvement

We did not involve patients or the public in the design, conduct, reporting, or dissemination of our research.

### Dissemination declaration

We do not plan to disseminate the results directly to study participants and or patient organisations.

## Results

Of the 766,244 singleton children born in Scotland who attended Scottish schools between 2009 and 2013, 4,788 (0.6%) had a history of being hospitalised for TBI. Of these, 3,381 (70.7%) were hospitalised for ≤1 day, 1,256 (26.2%) for 2 to 6 days, and 151 (3.2%) for ≥7 days. Overall, 2,965 (56.7%) children were hospitalised (or first hospitalised if hospitalised more than once) for TBI at <3 years of age, 858 (17.9%) at 3 to 6 years of age, 611 (12.8%) at 7 to 11 years of age, and 354 (7.4%) at 12 to 18 years of age. The mean age at first head injury admission was 3.73 years (median = 1.77 years). The mean length of follow up from first head injury varied by outcome measure; 9.44 years for assessment of SEN and 9.53, 12.70, and 13.74 years for absenteeism and exclusion, attainment, and unemployment, respectively.

Children previously hospitalised for TBI were significantly more likely to be white and male and were born in more deprived areas (Table 1). Their mothers were younger, less likely to be married, and more likely to have smoked during the current pregnancy and be parous (given birth previously). They were less likely to have had an operative delivery but were more likely to have been born preterm and had a lower birth weight for gestational age and sex. Demographic variables that contained the largest percentages of missing data included marital status (6.8%) and maternal smoking (11.4%). However, missing values for these variables were analysed as “unknown” categories and included in all analyses to minimise loss of records. The remaining variables were ordinal categories therefore including missing data as unknown categories did not make sense. However, the percentage of missing data within these variables was less than or equal to 0.2%, except for parity (0.5%), Apgar score (1.0%), and ethnicity (1.4%). Therefore, we did not deem multiple imputation to be necessary and instead used complete case analyses.

**Table 1:** Characteristics of study participants by whether or not they were previously hospitalised for TBI.

		No TBI *n* = 761,456		TBI *n* = 4,788		*P* value
		*n*	%	*n*	%	
Sex						
	Male	387,414	50.9	2,876	60.1	<0.001^1^
	Female	374,042	49.1	1,912	39.9	
Deprivation quintile category at birth						
	1 (most deprived)	204,304	26.9	1,749	36.6	<0.001^2^
	2	161,198	21.2	1,042	21.8	
	3	139,924	18.4	745	15.6	
	4	131,214	17.3	606	12.7	
	5 (least deprived)	123,040	16.2	636	13.3	
	Missing	1,776		0		
Ethnic group						
	White	722,718	96.2	4,585	97.3	<0.001^1^
	Asian	17,673	2.4	89	1.9	
	Mixed	6,679	0.9	27	0.6	
	Other	3,940	0.5	12	0.3	
	Missing	10,446		75		
Maternal age (years)						
	≤24	208,156	27.3	1,722	36.0	<0.001^2^
	25–29	223,055	29.3	1,485	31.0	
	30–34	215,853	28.3	1,082	22.6	
	≥35	114,380	15.0	499	10.4	
	Missing	12		0		
Marital status						
	Never married	218,813	30.9	1,610	34.4	<0.001^1^
	Married	369,144	52.1	2,414	51.6	
	Divorced/separated	3,631	0.5	65	1.4	
	Other	52,497	7.4	348	7.4	
	Not known	65,086	9.2	241	5.2	
	Missing	52,285		110		
Maternal smoking						
	No	488,544	72.4	2,560	63.2	<0.001^1^
	Yes	186,301	27.6	1,488	36.8	
	Missing	86,601		740		
Parity						
	0	343,595	45.4	2,070	43.3	<0.001^2^
	1	262,478	34.6	1,664	34.8	
	>1	151,524	20.0	1,046	21.9	
		3,859		8		
Mode of delivery						
	Spontaneous vaginal (cephalic)	512,809	67.3	3,410	71.2	<0.001^1^
	Operative/assisted vaginal (cephalic)	91,114	12.0	543	11.3	
	Vaginal breech	2,209	0.3	24	0.5	
	Elective cesarean	58,033	7.6	281	5.9	
	Emergency cesarean	97,126	12.8	530	11.1	
	Other/missing	164	0.0	0	0.0	
Gestational age at birth (weeks)						
	≤27	1,141	0.1	13	0.3	<0.001^2^
	28–32	6,995	0.9	63	1.3	
	33–36	35,358	4.6	244	5.1	
	37	37,352	4.9	267	5.6	
	38	95,371	12.5	621	13.0	
	39	157,752	20.7	948	19.8	
	40	228,952	30.1	1,478	30.9	
	41	170,256	22.4	937	19.6	
	≥42	27,683	3.6	211	4.4	
	Missing	556		6		
Sex- and gestation-specific birth weight centile						
	1–3	31,252	4.1	234	4.9	<0.001^2^
	4–10	68,191	9.0	455	9.5	
	11–20	90,781	11.9	567	11.9	
	21–80	447,297	58.8	2,824	59.1	
	81–90	65,002	8.5	361	7.6	
	91–97	40,970	5.4	251	5.3	
	98–100	16,992	2.2	87	1.8	
	Missing	971		9		
5-min Apgar						
	1–3	3,675	0.5	34	0.7	0.37^2^
	4–6	7,267	1.0	35	0.7	
	7–10	742,718	98.5	4,694	98.6	
	Missing	7,796		25		

^1^Produced using Chi square test for association.

^2^Produced using Chi square test for trend.

n, number; TBI, traumatic brain injury.

Overall, 116,845 (15.2%) pupils had a record of SEN: 56,288 (7.3%) due to learning difficulties; 34,774 (4.5%) social, emotional, and behaviour difficulties; 16,485 (2.9%) autism spectrum disorder; 22,292 (2.2%) learning disabilities; 10,814 (1.4%) communication problems; 9,177 (1.2%) physical health problems; 8,276 (1.1%) sensory impairment; 6,169 (0.8%) physical motor impairment; and 2,085 (0.3%) mental health problems. Among pupils with a history of hospitalised TBI, 933 (19.5%) had a record of any SEN compared with 115,313 (15.1%) of their peers.

An independent correlation structure was deemed most appropriate for the various GEE models. On univariate analysis, children with a history of hospitalised TBI were significantly more likely to have a record of SEN than their peers (OR 1.62, CI 1.50 to 1.75, *p* < 0.001). The effect size was attenuated, but remained significant, after adjusting for sociodemographic (OR 1.35, CI 1.25 to 1.47, *p* < 0.001) and maternity (OR 1.28, CI 1.18 to 1.39, *p* < 0.001) factors. Previous TBI hospitalisation was significantly associated with all causes of SEN except for learning difficulty, autism spectrum disorder, and mental health problems, and the association with SEN attributable to communication problems was of borderline significance. The effect sizes were largest for physical health problems and physical motor impairment (Table 2).

**Table 2:** Associations between previous hospitalisation for traumatic brain injury and special education need.

	Univariate			Partially adjusted*			Fully adjusted**		
	*N* = 766,244			*N* = 765,666			*N* = 701,307		
	OR	95% CI	*P* value	OR	95% CI	*P* value	OR	95% CI	*P* value
Any SEN	1.62	1.50–1.75	<0.001	1.35	1.25–1.47	<0.001	1.28	1.18–1.39	<0.001
Learning disability	1.81	1.55–2.13	<0.001	1.51	1.28–1.77	<0.001	1.42	1.21–1.67	<0.001
Learning difficulty	1.37	1.22–1.54	<0.001	1.13	1.01–1.28	0.04	1.07	0.95–1.21	0.26
Physical motor impairment	2.43	1.95–3.02	<0.001	2.21	1.77–2.76	<0.001	2.09	1.66–2.64	<0.001
Sensory impairment	2.06	1.54–2.75	<0.001	1.83	1.36–2.45	<0.001	1.72	1.28–2.31	<0.001
Communication problems	1.27	1.02–1.59	0.04	1.41	1.13–1.77	0.003	1.30	1.02–1.64	0.03
Autism-spectrum disorder	1.16	0.88–1.52	0.29	0.91	0.69–1.20	0.51	0.94	0.71–1.24	0.66
Social-emotional-behavioural difficulties	2.02	1.78–2.28	<0.001	1.59	1.40–1.80	<0.001	1.44	1.26–1.63	<0.001
Physical health	2.42	1.94–3.02	<0.001	2.05	1.64–2.56	<0.001	1.89	1.50–2.37	<0.001
Mental health	2.10	1.36–3.25	<0.001	1.33	0.86–2.05	0.20	1.20	0.77–1.87	0.42

*Adjusted for pupil age, sex, ethnic group, and Scottish Index of Multiple Deprivation quintile category.

**Also adjusted for maternal age, marital status, smoking status, parity, mode of delivery, gestational age at delivery, sex-/gestation-specific birth weight centile, and 5-min Apgar score.

CI, confidence interval; OR, odds ratio; SEN, special education need.

Absenteeism and exclusion data were available for 702,210 (91.6%) pupils. Children with a history of hospitalised TBI had a higher rate of absence than their peers on univariate analysis (IRR 1.39, CI 1.35 to 1.45, *p* < 0.001). Following adjustment for sociodemographic (IRR 1.15, CI 1.11 to 1.18, *p* < 0.001) and maternity (IRR 1.09, CI 1.06 to 1.12, *p* < 0.001) factors, the effect estimates were attenuated but were still statistically significant.

Children with prior hospitalised TBI incurred more exclusions from school than their peers. They had a higher rate of exclusion on univariate analysis (IRR 2.64, CI 2.27 to 3.06, *p* < 0.001) and after adjustment for sociodemographic (IRR 1.52, CI 1.31 to 1.76, *p* < 0.001) and maternity (IRR 1.33, CI 1.15 to 1.55, *p* < 0.001) factors, the effect sizes were attenuated but remained significant.

Examination attainment data were available for 139,205 (18.2%) pupils: 1,474 with previous TBI and 137,731 of their peers. Low attainment was recorded for 712 (48.3%) pupils with previous TBI compared with 49,679 (36.1%) of their peers. Low attainment was associated with TBI on univariate analysis (OR 1.66, CI 1.49 to 1.84, *p* < 0.001) and after adjustment for sociodemographic (OR 1.42, CI 1.22 to 1.65, *p* < 0.001) and maternity (OR 1.30, CI 1.11 to 1.51, *p* < 0.001) factors.

School-leaver destination was available for 217,924 (28.4%) former pupils. The average age on leaving school was 17.14 (median = 17.37) years among children with a TBI and 17.19 (median = 17.43) among peers. Among children previously admitted for a TBI, 336 (12.2%) left school before age 16 years compared with 21,941 (10.2%) of those not admitted for TBI. Of those with previous TBI, 339 (12.3%) were unemployed 6 months after leaving school, compared with 22,380 (10.4%) of their peers. Previous hospitalised TBI was associated with unemployment on univariate analysis (OR 1.21, CI 1.08 to 1.35, *p* < 0.001) but became nonsignificant after adjustment for sociodemographic (OR 1.08, CI 0.96 to 1.22, *p* = 0.19) and maternity (OR 1.03, CI 0.92 to 1.16, *p* = 0.61) factors.

Following exclusion of 989 children whose previous TBI was coded as concussion, the percentage of children with TBI who required hospitalisation for >1 day increased from 29.4% to 35.9% and the magnitude of the associations with previous TBI increased for SEN (fully adjusted OR 1.35, CI 1.23 to 1.48, *p* < 0.001), school absences (fully adjusted IRR 1.12, CI 1.08 to 1.16, *p* < 0.001), exclusion (fully adjusted IRR 1.39, CI 1.17 to 1.64, *p* < 0.001), and low attainment (fully adjusted OR 1.42, CI 1.19 to 1.68, *p* < 0.001). The association with unemployment was significant after adjusting for sociodemographic factors (OR 1.16, CI 1.02 to 1.32, *p* = 0.02) but remained nonsignificant following adjustment for maternity factors (fully adjusted OR 1.10, CI 0.97 to 1.25, *p* = 0.15).

## Discussion

Children previously admitted to hospital for TBI were more likely to have absences or exclusions from school. They were more likely to have a record of SEN due to learning disability, learning difficulty, sensory, physical or motor impairment, communication problems, physical health problems, or social, emotional or behavioural difficulties, and they were more likely to have low attainment in examinations.

It has previously been reported that 39% of children were enrolled in special education 1 year following hospitalisation for TBI [33] and that 50% to 79% had either failed a grade or been placed in special education 2 or more years after TBI [34,35]. Previous studies have suggested that the risk of SEN was greater among children who had severe TBI [33,35–40], which is consistent with our observation of stronger associations with adverse outcomes when hospital admissions for concussion were excluded. Our findings help to address the paucity of evidence on the association between childhood TBI and absenteeism and exclusion. Behavioural problems are well-recognised sequelae of childhood TBI [37,41–44], and a single UK study of 525 children admitted to hospital for TBI reported a dose-relationship between injury severity and parent-reported school absences and exclusions over 3-year follow-up [38]. Sociodemographic and maternity factors play an important role in predicting behavioural problems independent of childhood TBI. Indeed, previous studies have reported associations between maternity factors and educational outcomes including SEN [27–29]. Our study demonstrated that the observed associations between hospitalisation for TBI and later educational outcomes remained even after adjusting for maternity and sociodemographic factors.

Current evidence does not suggest that mild TBI in childhood results in significant or lasting reductions in school performance, but children with a moderate or severe TBI achieve significantly lower scores in the reading, mathematical, and language domains of achievement tests [35]. There is consistent evidence that deficits in academic achievement persist beyond the first-year post injury [43,45–48], and that, 2 years after severe TBI, children are 18-fold more likely to have an unfavourable academic performance than their peers [35].

Severe TBI in childhood is also associated with poorer academic performance in adulthood, compared with moderate or mild TBI or orthopaedic injuries [19,49,50]. Studies on the association between childhood TBI and employment are few in number and have reported inconsistent findings [19,49–51], but 2 studies have demonstrated a significant association between history of childhood TBI and receipt of government benefits [49,50].

Most previous studies of childhood TBI and educational outcomes have been small-scale. Indeed, only 2 previous studies have used administrative data. Gabbe and colleagues used the Welsh Electronic Cohort for Children to study 90,611 children born between 1998 and 2001 [52]. They reported that the 290 children previously hospitalised for TBI performed less well in their Key Stage 1 National Curriculum assessment, administered at 5 to 7 years of age, than their peers. Sariaslan and colleagues used a Swedish cohort of children born between 1973 and 1985, to compare 104,290 adults who sustained a TBI prior to 25 years of age with their 68,268 unaffected siblings [50]. Previous TBI was associated with not achieving secondary school qualifications, and receipt of means-tested welfare benefits and disability pension, as well as psychiatric disorders and premature mortality [50]. Jackson and colleagues analysed data from the USA National Survey of Children’s Health, on 15,402 preschool children (3 to 5 years) and demonstrated reduced school readiness among the 252 with a history of TBI [53].

The strengths of our study include its large, unselected sample size, recruitment of children from both mainstream and special schools, and the ability to investigate 6 educational outcomes in the same cohort. We were able to adjust for a range of sociodemographic and maternity confounders but, as with all observational studies, residual confounding is possible. Since both exposure and outcome data were collected routinely by the health and education sectors, recall or reporting bias was avoided. Association does not necessarily infer causality. However, the likelihood of the associations being causal was strengthened by the increased effect estimates observed when less severe traumatic brain injuries were excluded. Since SEN was recorded at school, for TBIs occurring before school age it was impossible to determine whether the underlying reason for subsequent SEN was already present before the TBI. Additionally, it is conceivable that, in some cases where the SEN was genuinely diagnosed after the TBI, the underlying difficulties necessitating SEN may have been present for a period beforehand and possibly before the TBI. Therefore, for the specific outcome of SEN reverse causation may be a limitation. It is also possible that not all children are formally identified as having SEN post TBI despite experiencing difficulties. Children born in private hospitals, privately educated, or home-schooled could not be included in the study. However, only 4% of Scottish children attend private schools [54], and home-schooling and private maternity care are very uncommon in Scotland. Because of the need to link birth and education records, we could not include children born outside of Scotland or who emigrated from Scotland before starting school. Finally, the large sample size contributed to small *p*-values (*<*0.001). Some of the findings, therefore, while highly statistically significant, may be less clinically significant since they represented modest differences in real terms, and this should be considered during interpretation.

Previous studies have demonstrated heterogeneity in outcome and recovery trajectory for childhood sufferers of TBI [16,17,55]. Our analyses could not investigate age at injury or time since injury which are further limitations and areas for potential future analyses. Future studies utilising population-level data sources present an opportunity for further investigation into the relationships between childhood TBI and educational outcomes accounting for additional factors not included in this study.

In conclusion, our findings demonstrated that childhood hospitalisation for TBI was associated with a wide range of adverse educational outcomes that reinforces the importance of preventing TBI where possible. Where not possible, children with a history of TBI should be supported to minimise the harmful effects incurred on their education.

## Supporting information

S1 STROBE Checklist STROBE Statement—checklist of items that should be included in reports of observational studies. (DOCX)

S1 Fig Flow diagram presenting the number of records and pupils excluded at each stage of data cleaning and data analyses. (TIF)

## Abbreviations

GEE: generalised estimating equation
ICD: International Classification of Diseases
SCQF: Scottish Credit and Qualifications Framework
SEN: special educational need
SIMD: Scottish Index of Multiple Deprivation
SQA: Scottish Qualifications Authority
TBI: traumatic brain injury
