# Supplementary figures and images for: Educational and employment outcomes associated with childhood traumatic brain injury in Scotland: A population-based record-linkage cohort study
Source: PLoS Med. 2023 Mar 28;20(3):e1004204. doi: 10.1371/journal.pmed.1004204 (PMC10047529; doi:10.1371/journal.pmed.1004204)

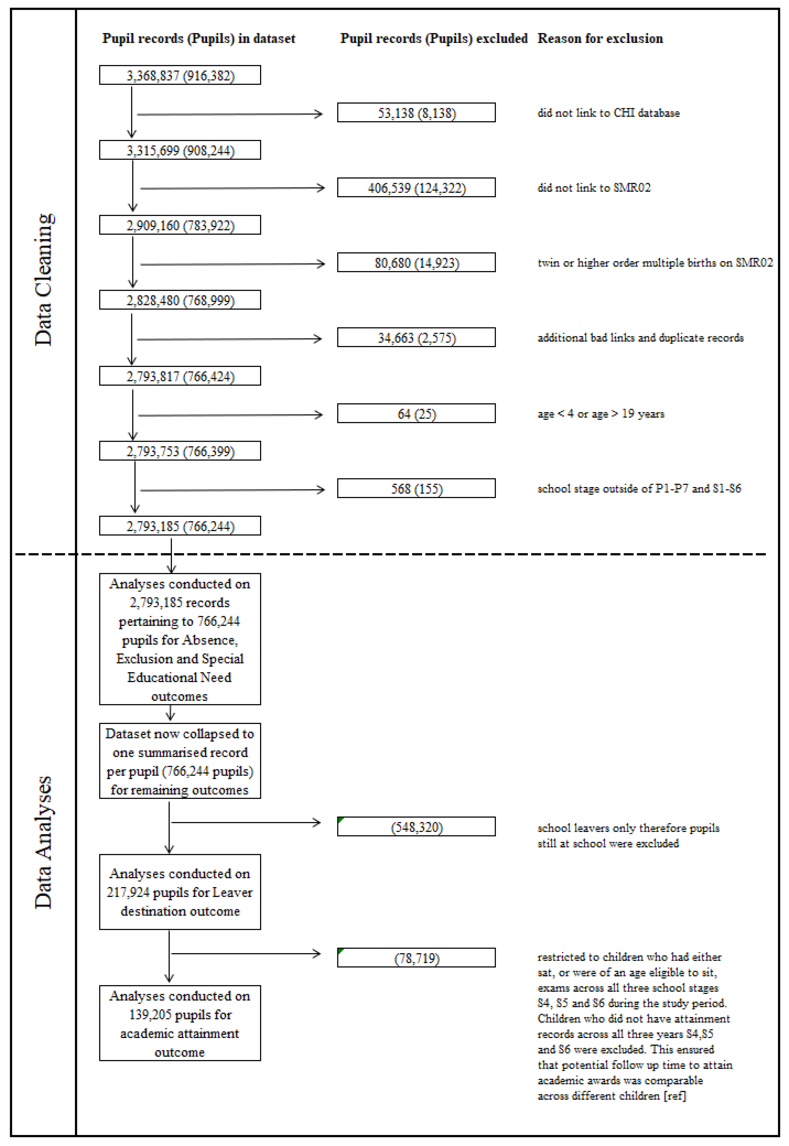

Supplement: S1 Fig — (TIF) [file pmed.1004204.s002.tif]
